# Courtship Pheromone Use in a Model Urodele, the Mexican Axolotl (*Ambystoma mexicanum*)

**DOI:** 10.1038/srep20184

**Published:** 2016-02-04

**Authors:** Margo Maex, Ines Van Bocxlaer, Anneleen Mortier, Paul Proost, Franky Bossuyt

**Affiliations:** 1 Amphibian Evolution Lab, Biology Department, Vrije Universiteit Brussel (VUB), Pleinlaan 2, B-1050 Brussels, Belgium; 2 Laboratory of Molecular Immunology, Department of Microbiology and Immunology, KU Leuven - University of Leuven, Minderbroedersstraat 10 - box 1030, B-3000 Leuven, Belgium

## Abstract

Sex pheromones have been shown to constitute a crucial aspect of salamander reproduction. Until now, courtship pheromones of Salamandridae and Plethodontidae have been intensively studied, but information on chemical communication in other urodelan families is essentially lacking. The axolotl (*Ambystoma mexicanum*, Ambystomatidae) has a courtship display that suggests a key role for chemical communication in the orchestration of its sexual behavior, but no sex pheromones have yet been characterized from this species. Here we combined whole transcriptome analyses of the male cloaca with proteomic analyses of water in which axolotls were allowed to court to show that male axolotls secrete multiple ca. 20 kDa glycosylated sodefrin precursor-like factor (SPF) proteins during courtship. In combination with phylogenetic analyses, our data show that the male cloaca essentially secretes a courtship-specific clade of SPF proteins that is orthologous to salamandrid courtship pheromones. In addition, we identified an SPF protein for which no orthologs have been described from other salamanders so far. Overall, our study advocates a central role for SPF proteins during the courtship display of axolotls and adds knowledge on pheromone use in a previously unexplored deep evolutionary branch of salamander evolution.

The Mexican axolotl (*Ambystoma mexicanum*) is an aquatic, neotenic mole salamander with a long and fruitful history in scientific research. Axolotls have been serving as valuable, non-traditional model organisms for developmental biology since the middle of the 19^th^ century^1^,^2^ and are still extensively used in laboratory experiments, covering research topics such as regeneration, development, neuroscience and olfaction^3^,^4^. Since axolotls have been bred in laboratories for over 150 years^2^, observational records on their reproductive behaviour are readily available^4^,^5^,^6^,^7^. Axolotls display a courtship dance that suggests a key role for pheromones in the orchestration of their sexual behaviour. Nonetheless, surprisingly little is known about the chemical ecology of this model organism, except for its ability to determine the reproductive status and sex of conspecifics based on chemical cues^8^.

Mole salamanders (Ambystomatidae) belong to the internally fertilizing salamanders and similar to most members of this clade, they do not engage in copulation to reproduce^7^. Instead, a male transfers his sperm to a female through a spermatophore deposited in the environment. In order to attain internal fertilization, females are persuaded to pick up this sperm package with their cloaca during an elaborate courtship display. Male axolotls vigorously nudge the female with their snout and perform a so-called ‘hula dance’ in which they widely open their cloaca and undulate the posterior parts of their body and tail. The female responds to the courting male by nudging the male’s cloacal region. When the male moves forward and deposits a spermatophore, the female follows him and picks up the sperm cap with her cloaca^4^,^5^,^6^,^7^. Courting males are believed to secrete chemical cues in the water when they widely open their cloaca and expose the cloacal papillae to the female. The sexually dimorphic cloaca of a male axolotl harbours six different types of glands, including the vent gland which is assumed to be the pheromone-producing gland^9^. Moreover, the strong interest of a female in a male’s cloaca emphasizes the importance of chemical communication during courtship. Despite clear indications that sex pheromones affect axolotl reproductive behaviour, knowledge on the nature of these molecules in the axolotl, and ambystomatids in general, is largely absent.

Over the past 20 years, research on sex pheromones in salamanders has led to the discovery of three unrelated protein pheromone systems in two distinct urodelan families (Plethodontidae and Salamandridae)^10^,^11^,^12^,^13^,^14^. Only proteins of the highly diversified Sodefrin Precursor-like Factor (SPF) pheromone system were shown to fulfil a courtship function in both salamandrid and plethodontid species^12^,^13^. In the terrestrial plethodontid *Desmognathus ocoee*, fractions enriched with 23 kDa SPF proteins were shown to shorten the duration of courtship when applied to the dorsal skin of a female^12^. In a natural courtship sequence of this species, males scratch the back of the female with their premaxillary teeth and rub their mental gland over the newly-formed skin abrasion to ‘vaccinate’ pheromones into the female’s blood stream and increase her receptivity^15^. In the aquatically reproducing salamandrid *Lissotriton helveticus* (palmate newt), males fan an array of 21 kDa SPF proteins towards the female with their tail during courtship^13^. Two-female behavioural experiments showed that a single SPF isoform is sufficient to induce stereotyped female sexual behavioural responses. In addition, the seemingly unrelated decapeptide sodefrin, which exhibits a female-attracting activity in the Japanese fire-bellied newt *Cynops pyrrhogaster* (Salamandridae)^16^, was shown to have originated through a frameshift in one of the SPF genes^17^. Irrespective of the wide variety in courtship strategies and pheromone delivery modes, the SPF pheromone system seems to have retained a central role during courtship of internally fertilizing salamanders.

Mole salamanders (Ambystomatidae) diverged from Salamandridae about 150 million years ago (Mya)^18^ and therefore constitute a long branch of independent evolutionary history in amphibian evolution. Here we combined cloacal gland transcriptome data and protein characterisation of water in which axolotls were allowed to court, with phylogenetic analyses to gain insight in axolotl (*Ambystoma mexicanum*) courtship pheromone use. Proteomic analyses showed that courting male axolotls secrete multiple ca. 20 kDa glycosylated SPF proteins in the water. The combination of these proteomic data with transcriptome analyses allowed identification of the full-length precursor transcripts, the corresponding proteins they encode and their individual expression levels in the male cloaca. Finally, we combined a gene phylogeny of known salamandrid and plethodontid SPF precursors with axolotl pheromone candidates to demonstrate their orthology with established urodelan courtship pheromones.

## Results and Discussion

### Axolotls actively secrete SPF proteins during courtship

**Figure 1:**
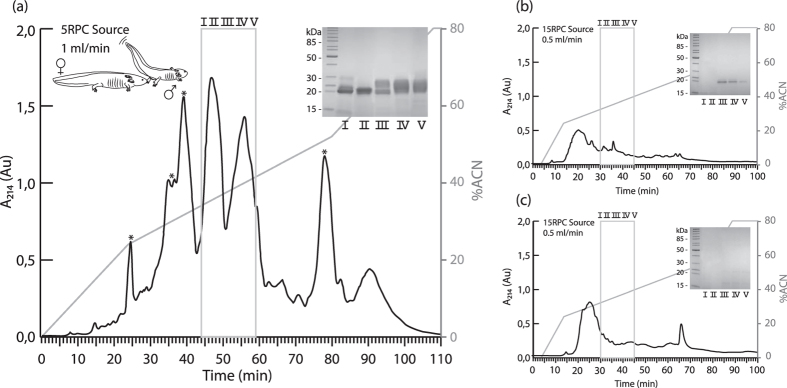
Chromatographic separation of protein content in courtship, male and female water. **(a)** RP-HPLC elution profile of courtship water (n = 5 courting couples) shows several courtship-specific peaks that are absent in male and female water. A silver-stained SDS-PAGE gel of fractions I to V reveals the presence of multiple 20–25 kDa proteins. **(b)** RP-HPLC elution profile of male (n = 5) and **(c)** female (n = 5) water with silver-stained SDS-PAGE gel. A 20 kDa band appears in fractions III to V of male water. The acetonitrile gradient (0% to 80% ACN in 0.1% TFA) is represented by a grey line in all elution profiles.

To investigate what molecules are secreted from a male’s cloaca during courtship, we collected water in which axolotls were allowed to court (hereafter referred to as ‘courtship water’), and compared this with both water in which non-courting males had been held and water in which non-courting females had been swimming. Proteins were directly extracted from the three types of water and further separated on a reversed phase high-performance liquid chromatography (RP-HPLC) system. Our analyses indicate that the RP-HPLC elution pattern of courtship water differs profoundly from water in which non-courting animals had been held (Fig. 1a–c), evidencing that some molecules were specifically released during courtship. A direct protein comparison was made between RP-HPLC purified male, female and courtship water using mass spectrometry, gel-electrophoresis and N-terminal protein sequencing by Edman degradation. Gel electrophoresis of the main differential peak shows the presence of several 20–25 kDa proteins (Fig. 1a). At similar solvent concentrations, a faint 20 kDa band is visible in male water (Fig. 1b), while no such band is apparent in female water (Fig. 1c, see also Supplemental Figure S3). Edman degradation sequencing shows that, during courtship, at least seven different 20–25 kDa proteins are secreted in the water that bear a distinctive N-terminal cysteine pattern typical for SPF proteins (Table 1). These findings suggest that, similar to salamandrids and plethodontids, axolotls augment female receptivity during courtship by secreting a blend of highly distinct SPF proteins. Sequencing by N-terminal degradation of other molecules in courtship-specific differential peaks (Fig. 1a, indicated with an asterisk) revealed that these do not belong to the SPF pheromone system (data not shown). Conversely, N-terminal sequencing of the faint 20 kDa band in male water revealed an SPF protein whose amino acid sequence is identical to one of the SPF proteins present in courtship water (Table 1, sequences corresponding to SPF12 and SPF13). No N-terminal sequences could be generated that confirm the presence of SPF proteins in female water. Finally, mass spectrometry identified a series of glycoproteins in courtship and male water that could be matched to the theoretical relative molecular mass (Mr) of *in silico* translated transcripts obtained by RACE-PCR (see Supplementary note and Table S1), while no SPF masses were measured in the relevant fractions of female water.

### Male axolotl SPFs are orthologous to established pheromones

**Figure 2:**
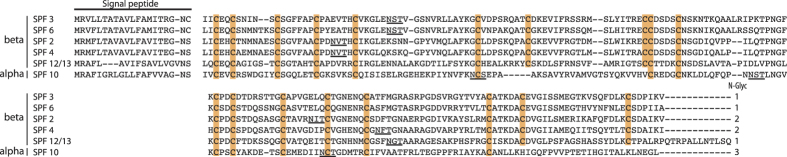
Amino acid sequences and conserved cysteine patterns of translated SPF cDNA precursors in *A. mexicanum*. MAFFT alignment of a selection of SPF precursors expressed in male cloacal tissue shows the presence of a cysteine pattern typical for alpha and beta SPFs (cysteines are indicated in orange). Predicted N-glycosylation sites are underlined and the number of predicted sites per isoform is indicated.

To further characterize the axolotl SPF pheromone candidates, we performed RACE-PCR with a diverse set of primers (see Methods). Thirteen different SPF cDNA precursor sequences were identified this way, encoding 12 unique mature SPF proteins, each with a 20 AA signal peptide (SPF1-13, GenBank numbers KU043451-KU043463). Earlier studies have differentiated two types of SPF, both encoding for a two-domain three-finger protein, but exhibiting a distinct cysteine pattern: alpha SPFs show an anterior 8-cysteine motif and a posterior 6-cysteine motif, while beta SPFs have an anterior 10-cysteine motif followed by a posterior 8-cysteine motif^17^. Molecular dating estimates suggest that the gene duplication that gave rise to these SPFs happened near the origin of stem salamanders about 300 Mya^13^. In our transcripts, two highly similar SPF precursors (SPF10 and SPF11) exhibit a cysteine pattern typical for alpha SPF, while all other precursors show a beta SPF cysteine pattern (Fig. 2). Thus, descendants of both anciently diverged SPF copies are currently expressed in the cloaca of male axolotls.

**Figure 3:**
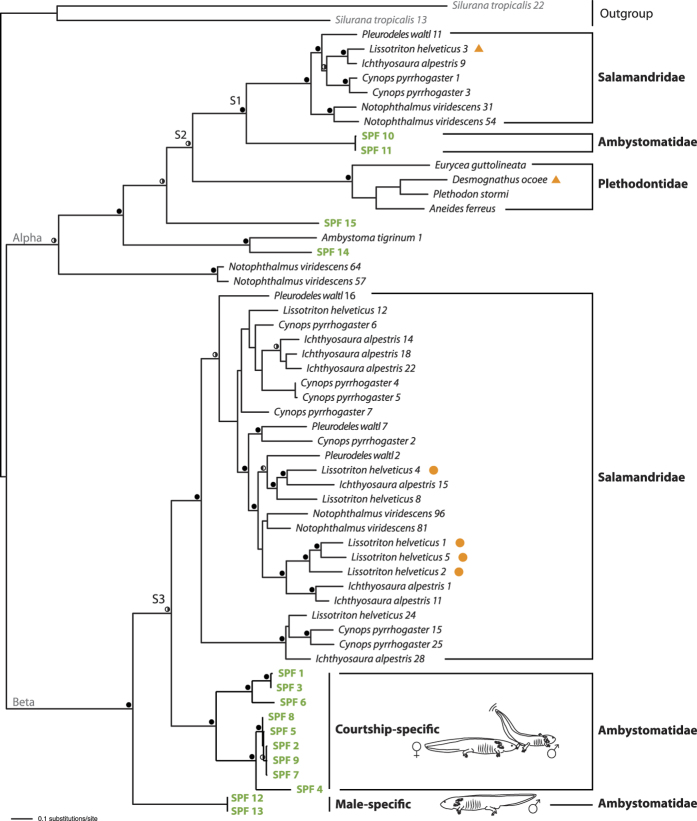
Maximum likelihood tree of SPF protein diversification. Filled circles on the branches indicate ML bootstrap support equal to or higher than 70 in combination with Bayesian posterior probabilities (BPP) of 0.95 or higher. Half-filled circles indicate support for BPP alone (0.95 or higher, left black) and ML-bootstrap support alone (70 or higher, right black). Nodes S1 and S3 represent the Ambystomatidae-Salamandridae speciation event and node S2 the [Ambystomatidae-Salamandridae]-Plethodontidae split, as confirmed by Notung analyses. *Ambystoma mexicanum* SPF sequences are indicated in bold green. The ‘courtship-specific’ clade is comprised of SPF isoforms that are exclusively secreted in courtship water, as confirmed by Edman degradation sequencing. SPF12 and SPF13 represent male-specific SPFs likely not originating from the cloaca. Orange dots and triangles show SPF protein sequences that have previously been identified as courtship pheromones^12^,^13^.

To determine the phylogenetic position of the identified axolotl SPF precursors in relation to known salamandrid and plethodontid SPF precursors, we combined the mature coding sequences of our axolotl SPF precursors with precursor sequences available on GenBank for salamandrids^13^,^17^ and plethodontids^19^. Two frog sequences were selected as outgroup^17^ (Supplementary Table S2). Alignment resulted in a data matrix of 56 Operational Taxonomic Units (OTUs) and 239 characters, for which ProtTest 2.4^20^ assigned JTT + G as the best fitting model of amino acid replacement under the Akaike Information Criterion (AIC). Maximum Likelihood and Bayesian analyses revealed a single best tree (-Ln *L* = 12277.47) (Fig. 3) that is largely congruent with previously published SPF gene trees^13^,^17^,^21^. Furthermore, our tree allows unambiguous identification of the speciation events that represent the family level diversifications in both alpha and beta SPF (Fig. 3, node S1 to S3). Within alpha SPFs, our tree strongly supports orthology of two ambystomatid precursors (Fig. 3, SPF10 and SPF11) with salamandrid and plethodontid SPFs containing demonstrated courtship pheromones^12^,^13^ (Fig. 3, orange triangles). Within beta SPFs, a clade of diverse precursors with pairwise amino acid divergences of up to 42% (Fig. 3, ‘courtship-specific’) originated through lineage-specific duplications that occurred after the Ambystomatidae-Salamandridae split (Fig. 3, node S3). This clade is orthologous to the highly diversified beta salamandrid SPF clade, for which multiple isoforms were shown to be tail-fanned by palmate newt males as courtship pheromones^13^ (Fig. 3, indicated with orange dots). So far, no orthologs of the remaining sequences (SPF12 to SPF15) were identified in the pheromone-producing glands of urodelans^13^,^17^,^21^,^22^.

### Courtship-specific beta SPFs are highly expressed in the male cloaca

To estimate the relative expression of SPF precursors amplified from male cloaca tissue, we performed transcriptome sequencing (RNASeq) of the cloacal glands of a sexually active male axolotl, yielding 127,404,240 paired-end (100 bp) reads. Expression analyses in CLC Genomics Workbench (v8.0.1) on *de novo* assembled SPF transcripts (including 5′ and 3′-UTR) revealed 3,557,293 reads belonging to the SPF family, indicating that about 2.8% of all reads belong to the SPF protein family. Subsequent expression analyses on the coding region of transcripts obtained by RACE-PCR resulted in mapping of 1,153,834 reads, and indicate that a strongly supported clade of beta SPFs jointly account for nearly all SPF expression (99.2%) in the male’s cloaca (Table 2, SPF1 to SPF9; Fig. 3, ‘courtship-specific’). The presence of the corresponding proteins in courtship water is supported by several Edman degradation sequences (Table 1). Together, the orthology with highly expressed salamandrid pheromones, the high transcript expression in the male cloacal glands of the axolotl, and the exclusive secretion during male courtship strongly advocate a female-receptivity enhancing pheromone function for this clade of beta SPFs. In contrast, we observed a low cumulative expression of 0.7% for the alpha SPF precursors (Table 2, SPF10 and SPF11), which is in line with Edman degradation sequencing signals being close to the background level. Though present in courtship water, the concentration of alpha SPFs is probably significantly lower than the beta SPFs, and their relative role as courtship pheromones in the male SPF blend may be limited.

With a relative expression of 0.08%, our transcriptome analyses suggest that SPF12 and SPF13 are hardly expressed in the cloaca (Table 2). This is remarkable, since the corresponding protein is present in both courtship water and male water. We currently cannot exclude that the discrepancy between proteome and transcriptome level expression is due to an exceptional translational efficiency or longevity of transcripts^23^,^24^. However, we consider it more likely that the cloaca is not the main organ secreting these SPFs, and that there is a continuous - i.e. not exclusively linked to courtship activities - passive release of the molecule from sexually mature males, potentially from epidermal skin secretion. Since protein synthesis carries a high energetic cost, this secretion may nevertheless be an essential part of the chemical communication system of axolotls. The male-specific SPF12/13 could be involved in sex identification, provide information on reproductive condition, or augment a female’s receptivity. Indeed, female axolotls were shown to perform courtship displays when exposed to whole-body odorants from males^8^ (i.e. male water). Since SPF12/13 is an important component of a male’s odorant blend, it will be interesting to examine how sexually mature females react on this particular SPF protein in a behavioural assay (e.g. similar to the one performed by Park *et al*. 2004^8^).

## Conclusions

Although chemical communication is generally acknowledged in the Mexican axolotl, ambystomatid pheromones had remained entirely unexplored. Here, we used combined transcriptomic and proteomic evidence to identify a set of male courtship-specific SPF proteins, further supporting the widespread presence of the SPF pheromone communication system in internally fertilizing salamanders^13^. In addition, we identified an SPF protein for which no orthologs have been described yet from other salamanders. This gene, which originated through a duplication more than 150 million years ago, is abundant in water containing sexually mature males. Behavioural experiments in which female axolotls are exposed to the identified SPF pheromone candidates are a prerequisite to achieve a more comprehensive understanding on the exact function of these SPF molecules in ambystomatid reproduction. Since axolotls were proposed as useful model organisms to study olfaction and the neurobiology of chemosensory systems, our findings will facilitate further research on pheromone perception. Electro-olfactogram recording techniques for investigating pheromone responses in the olfactory and vomeronasal epithelia of adult axolotls are readily available^4^,^8^. Electrophysiological experiments with female axolotls would enable us to study electro-olfactogram responses on each individual SPF, giving us insight on their relative contribution in the multicomponent putative pheromone blend. Finally, future research using demonstrated SPF pheromones could shed light on the function of the vomeronasal system relative to the olfactory system in pheromone processing^4^.

## Methods

### Animal husbandry

Eight males and six females were obtained from a commercial supplier (Petco, Deurne, Belgium), and raised until fully sexually mature. Sexual maturity was confirmed by spermatophore deposition or egg laying. Animals were kept in biologically filtered water in an air-conditioned room at 18 ± 1 °C under a light/dark regime of 12/12h. Males were housed individually in plastic tanks (l60 × h35 × w35 cm) filled with 35 l aged tap water, whereas females were housed per three (l78 × h50 × w60 cm with 150 l aged tap water). Axolotls were fed with earthworms (*Eisenia hortensis*), sturgeon pellets, thawed bloodworms (*Chironomidae* spp.) or fish (*Osmerus eperlanus, Pollachius pollachius*) two to three times a week.

### Ethics statement

One adult male was anaesthetized by immersion in 0.5 g/L buffered MS-222 (Sigma-Aldrich) and then euthanized by decapitation and pithing of the brain and spinal canal. This procedure does not violate any European convention (European Convention for the protection of Vertebrate animals used for experimental and other scientific purposes; CETS #123), Belgian law (Art. 2.6 of the Belgian Law of May 4th 1995; Annex VII, Belgian Law of May 29^th^ 2013), or institutional regulation. This research was approved by the Ethical Committee for Animal Experiments of the Vrije Universiteit Brussel (Project number 14-220-35), and all experiments were performed in accordance with the approved guidelines and regulations.

### Protein analyses

#### Collection, extraction and crude separation of molecules present in courtship, male and female water

For the collection of male and female-specific proteins, five sexually mature males and five sexually mature females each were kept separately in a plastic container (l34 × h12 × w19 cm) filled with 2 l of aged tap water for a period of 90 minutes. After 90 minutes, animals were returned to their housing containers and water of all same-sex individuals was pooled for further downstream analyses. To collect courtship water, a sexually mature couple was placed in a larger plastic container (l37 × h15 × w26 cm) filled with 4 l of aged tap water. Since courtship pheromones are assumed to be secreted from a male’s vent gland in the cloaca^9^, we measured the time that a courting male held his cloaca wide open. Only courtship water in which a male had opened its cloaca for 30 to 60 minutes was sampled and retained for protein extraction, as this stretch of time ensured a detectable amount of courtship proteins. For each proteomic downstream analysis, a minimum of five courting couples was combined (this was repeated three times in total). Male water, female water and courtship water were systematically collected in the dark using a dim light to monitor the animals’ behaviours and courtship sequences.

Before the actual protein extraction, water was filtered using 5 μm Durapore PVDF filters (SVLP04700, Millipore) to prevent column clogging. Molecules present in the three types of water were extracted using two kinds of reversed-phase adsorbent resins, viz. RP-C8 and RP-C18 (Sep-Pak plus cartridge, 400 mg sorbent, Waters) and each cartridge was loaded with a maximum of 400 ml water. Elution of molecules from these reversed-phase columns was carried out by applying 7.5 ml of 90% (v/v) acetonitrile (ACN) with 0.1% TFA. Removal of ACN from the eluate was achieved by evaporation in a Speedvac concentrator (SCV-100H, Savant instruments, Farmingdale, NY) for 1h. Concentrated samples of courting couples were pooled and loaded onto a Source 5RPC column (4.6 × 150 mm, GE Healthcare Life Sciences) pre-equilibrated with 0.1% (v/v) TFA (solvent A) to conduct reversed-phase high-performance liquid chromatography (RP-HPLC). After loading, the column was washed for 10 min at a constant flow rate of 1 ml per minute using solvent A. Proteins were eluted with 80% ACN in 0.1% TFA (solvent B) at 1 ml per minute as follows: 0–30% B in 24 minutes, 30–65% B in 56 minutes (increase of 0.5% ACN per minute) and lastly 65–100% B in 10 minutes. Since the 5RPC SOURCE column was no longer commercially available at the time male and female water was collected for purification, a 15RPC SOURCE column (4.6 × 100 mm, GE Healthcare Life Sciences) was used at a constant flow rate of 0.5 ml per minute. Proteins extracted from male and female water were eluted with 80% ACN in 0.1% TFA (solvent B) at 0.5 ml per minute by applying the following gradient: (1) 0–30% B in 10 minutes, (2) 30–70% B in 64 minutes (increase of 0.5% ACN per minute), (3) 70–100% B in 10 minutes. Absorbance of the eluting samples was measured at a wavelength of 214 nm and eluates were collected in 1 ml fractions. To visualize the protein content of male, female and courtship water, peak fractions were subjected to non-reducing SDS-PAGE using precast gels (Any kD Mini-PROTEAN TGX, Bio-Rad) and silver-stained (Silverquest Silver Staining kit, Invitrogen).

To ascertain all relevant proteins elute in a similar fashion on both the 15RPC and 5RPC column, courtship water samples were re-run on the 15RPC SOURCE column at a flow rate of 0.5 ml per minute applying the same gradient as used for the chromatographic separation of proteins in male and female water. The order in which the proteins of interest eluted did not change as a result of using a different column or flow rate. By re-running courtship water on the 15RPC column, we were able to define the entire SPF elution range that had to be screened to identify any potential SPF molecules in male and female water (see Supplementary Figure S3).

#### Mass spectrometry and amino acid sequence analyses

After separation by SDS-PAGE, proteins were transferred onto a polyvinylidene difluoride membrane by semi-dry blotting (Trans Blot Turbo System, Bio-Rad) and stained with 0.1% Coomassie brilliant blue R-250 (Sigma). Bands of interest were excised from the blot, destained with methanol and the amino acid sequence of the proteins determined on a 491 Procise cLC protein sequencer (Applied Biosystems). Proteins in 30 ul aliquots of each fraction were concentrated to 15 μl and 5 μl was further separated in a 0.1% formic acid to 80% ACN 0.08% formic acid gradient at 0.8 μl per minute on a C4 Acclaim Pepmap 5 μm (300 μm × 150 mm) column using an Ultimate 3000 RSLCnano system (Thermo Scientific) coupled to an amaZon speed electron transfer dissociation (ETD) ion trap (Bruker) for mass spectrometry analyses with a target mass of 1700 m/z. Each nano LC run was repeated with a target mass of 2500 m/z to detect proteins with a higher Mr and less charges. Averaged profile spectra of proteins were obtained using Bruker Daltonics deconvolution software (data analysis 4.1). The experimentally obtained average Mr of our SPF protein candidates was compared to the theoretical average Mr of all SPF cDNA precursors found in male cloacal tissue.

SDS-PAGE, mass spectrometry and Edman sequencing results in the main article are shown for compound fractions, each representing a set of three consecutive 1 ml fractions from the Source RPC columns. Compared fractions differ in elution range and thus fraction number due to the use of a different flow rate and column, and because of a faster initial gradient for the elution of male and female molecules (0–30% B in 10 min instead of 24 min, which did not affect the elution of SPF proteins, since these elute at higher ACN concentrations, see also Supplemental Figure S3). Mass spectra obtained per glycoform for each of the three consecutive 1 ml fractions were averaged.

#### Computation of theoretical molecular weight from SPF precursors

SPF cDNA precursors obtained by RACE-PCR (see Collection of transcriptome data) were translated into proteins using the ExPASy Translate tool^25^. Signal peptides were predicted using SignalP 4.1^26^ and N-glycosylation sites on the Asn-Xaa-Ser/Thr sequons were predicted with NetNGlyc 1.0^27^. To calculate the theoretical molecular mass of the SPF precursors, signal peptides were removed and protein sequences were entered in the pI/Mr tool on ExPASy^25^. Next, formation of all disulfide bridges was taken into account, and 2 N-acetylhexosamine units and 3 to 9 hexose residues were added to this mass (HexNAc = 203.19 Da, hexose = 162.14 Da). In case multiple N-glycosylation sites were predicted, additional calculations were made with two or three N-linked glycan trees.

### Transcriptome analyses

#### Collection of transcriptome data

Total RNA was extracted from cloacal tissue of a mature, sexually active male using TRI Reagent (Sigma-Aldrich) and the RNAeasy mini kit (Qiagen). *De novo* transcriptome sequencing was performed at DNAvision (Gosselies, Belgium). A paired-end PE100 cDNA sequencing library was constructed using the Illumina TruSeq RNA sample preparation kit for sequencing on the Illumina HiSeq2000 platform (Illumina, San Diego, California). Sequences in FASTQ format were obtained after filtering low quality reads, trimming of low quality sequence ends and trimming of adapters. A *de novo* assembly was generated in Trinity^28^ (r20140717 with the following parameters: min_per_id_same_path 99, max_diffs_same_path 1, max_internal_gap_same_path 3). SPF sequences were identified by aligning *de novo* to a dataset containing known palmate newt SPF sequences (GenBank numbers KJ402326–KJ402357) using RAPSearch^29^ and the BLAST application in CLC Genomics Workbench (v8.0.1).

Rapid amplification of cDNA ends (RACE) was performed to obtain complete protein sequences from different SPF precursors. Primers were designed on the 3′-untranslated region of all *de novo* assembled SPF transcripts for which the N-terminal amino acid sequence corresponded to a retrieved Edman degradation sequence. The following primers were used to amplify full-coding sequences of SPF transcripts: SPF A 5′-TGCACACTAACAATAGTACTGCTGC-3′, SPF B 5′-GCAGCAATACTACTCCTAATTACTATGC-3′, SPF C 5′-GCAGTAGGACTATTGCGTGTGTG-3′, SPF D 5′-CCGCAAGCTTCATACATGC-3′, SPF E 5′-GGGTACTACTGCTGACTACGATGC-3′ and SPF F 5′-ATGAGTGCACAGTAGGAAAGGC-3′. One microgram of total RNA from the same extraction procedure as obtained for RNASeq was used to construct RACE-ready cDNA with the SMARTer-RACE cDNA amplification kit (Clontech). FastStart High Fidelity Taq DNA polymerase (Roche) was used to amplify the PCR products. PCR products were purified using the Qia-Quick PCR purification kit (Qiagen) and used for cloning into a pGEM-T Easy cloning vector (Promega). Ligation products were transformed into TOP10 Competent Cells (Invitrogen), which in turn were grown overnight on LB-agar plates with Ampicillin and X-Gal. Apparent successfully cloned colonies were picked randomly and their inserts were amplified with Faststart Taq DNA polymerase. The obtained amplification products were cycle sequenced using the BigDye Terminator v3.1 Cycle Sequencing kit and visualized on an ABI Prism 3100 Genetic Analyzer (Applied Biosystems). Nucleotide sequences were assembled into contiguous sequences (contigs) with CodonCode Aligner v. 3.7.1.1 (CodonCode Corporation) and translated into proteins using the ExPASy Translate tool^25^.

#### Expression analyses

Transcript expression levels of *de novo* assembled transcripts as well as RACE-PCR cDNA precursors were estimated by mapping reads using the RNASeq module of CLC Genomics Workbench (v8.0.1), allowing only reads to map with a sequence similarity of 95% for 90% of the length of the read, resulting in highly specific mapping. Expression values are reported in RPKM (reads per kilobase of transcript per million mapped reads). To determine the expression of each transcript relative to all SPF transcripts, individual RPKM values were divided by the sum of all SPF transcript RPKM values.

### Phylogenetic reconstructions

We combined translated amino acid sequences of 13 SPF precursors obtained in the current study from *A. mexicanum* with SPF precursors from four plethodontid, three additional ambystomatid and 35 salamandrid sequences from Genbank (Supplementary Table S2). Two frog Phospholipase A2 Inhibitor (PLI) sequences were included as outgroup^17^. Amino acid sequences were aligned using the automatically assigned L-INS-I algorithm and standard automatic parameters implemented in Mafft v7^30^. ProtTest 2.4^20^ was used to select the best fitting model of amino acid (AA) replacement for this data set according to an AIC. Maximum-likelihood (ML) analyses were run in PAUP*^31^ using the ProtTest-assigned JTT + G model. Bayesian analyses were run with a mixed prior for the AA substitution model and gamma correction for among-site rate heterogeneity in MrBayes 3.2.2^32^ at the CIPRES Science Gateway^33^. Two parallel runs of four Markov chain Monte Carlo were executed for 10,000,000 generations, with trees sampled every 1,000^th^ generation and the first 5,000 generations discarded as burn-in. Convergence of parallel runs was confirmed by split frequency standard deviations (less than 0.01) and potential scale reduction factors (approximating 1.0) for all model parameters. Adequate posterior sampling for each run was verified using Tracer 1.6^34^, by examining the effective sampling sizes of all model parameters. Clade support under ML was assessed by 1,000 replicates of rapid bootstrapping using RAXML 7.0.4^35^ on the CIPRES Science Gateway v3.3^33^. Speciation-duplication analyses were performed in NOTUNG 2.6^36^,^37^,^38^.

## Additional Information

**How to cite this article**: Maex, M. *et al*. Courtship Pheromone Use in a Model Urodele, the Mexican Axolotl (*Ambystoma mexicanum*). *Sci. Rep*. **6**, 20184; doi: 10.1038/srep20184 (2016).

## Supplementary Material

Supplementary Information

## Figures and Tables

**Table 1:**
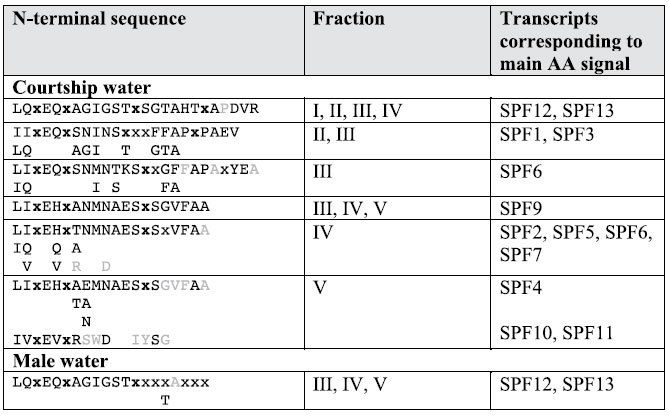
Identification of SPF proteins in axolotl courtship and male water.

N-terminal sequences were obtained by Edman degradation. Sequences of the 20-25 kDa protein bands exhibit a cysteine pattern (indicated in bold as 'x') typical for SPF proteins. The x's designate residues for which no signal was detected and usually represent unmodified cysteines that cannot be detected by Edman degradation (cysteines were not alkylated prior to the sequence analysis). Low sequencing signals close to the level of background are indicated in grey. Transcripts obtained by RACE-PCR that correspond to one of the N-terminal sequences are indicated.

**Table 2:** SPF transcript expression in the cloacal glands of male *A. mexicanum*.

**Precursor name**	**Gene length**	**RPKM value**	**Unique gene reads**	**Total gene reads**	**Relative SPF expression (%)**
**SPF 1**	606	314,484.6	115,913	219,895	19.15
**SPF 2**	609	233,157.1	79,980	163,836	14.20
**SPF 3**	609	232,928.0	86,470	163,675	14.18
**SPF 4**	609	229,253.5	161,012	161,093	13.96
**SPF 5**	609	204,182.6	72,954	143,476	12.43
**SPF 6**	615	169,579.6	120,335	120,335	10.33
**SPF 7**	609	107,809.3	26,748	75,756	6.57
**SPF 8**	609	89,765.7	25,759	63,077	5.47
**SPF 9**	609	47,820.9	11,438	33,603	2.91
SPF 10	594	7,509.7	3,911	5,147	0.46
SPF 11	594	3,431.7	1,719	2,352	0.21
SPF 12*	642	715.5	248	530	0.04
SPF 13*	642	666.9	234	494	0.04
SPF 14	594	440.6	302	302	0.03

Expression levels of transcripts are shown as reads per kilobase per million mapped reads (or RPKM values) and transcripts are ranked accordingly. Names in bold represent all courtship-specific beta SPFs. Asterisks depict transcripts with an identical protein product.
