# Supplementary material for: Courtship Pheromone Use in a Model Urodele, the Mexican Axolotl (Ambystoma mexicanum)
Source: Sci Rep. 2016 Feb 4;6:20184. doi: 10.1038/srep20184 (PMC4740750; doi:10.1038/srep20184)
Supplement: Supplementary Information [file srep20184-s1.pdf]

Courtship Pheromone Use in a Model Urodele, the Mexican Axolotl (*Ambystoma mexicanum*)

Margo Maex[1], Ines Van Bocxlaer[1], Anneleen Mortier[2], Paul Proost[2] & Franky Bossuyt[1]

[1] Amphibian Evolution Lab, Biology Department, Vrije Universiteit Brussel (VUB), Pleinlaan 2, B-1050 Brussels, Belgium

[2] Laboratory of Molecular Immunology, Department of Microbiology and Immunology, KU Leuven - University of Leuven, Minderbroedersstraat 10 - box 1030, B-3000 Leuven, Belgium

Contains:

Supplementary note: Glycoprotein identification.

Table S1: SPF glycoforms identified by mass spectrometry in courtship and male water.

Table S2: Accession numbers of SPF precursor sequences of species used in the phylogenetic analysis.

Supplementary Figure S3: Chromatographic separation of proteins present in courtship, male and female water using a 15RPC column.

Supplementary note: Glycoprotein identification.

To obtain the average relative molecular mass (Mr) of the secreted 20-25 kDa SPF proteins, molecules were separated by nano reversed-phase liquid chromatography and masses were determined by on-line mass spectrometry. Deconvolution of the obtained mass spectra revealed proteins that differ by the mass of one hexose residue (162-164 Da) from one another. A recent study identified similar series of glycoforms in the courtship pheromone blend of palmate newts[1]. Here SPFs were shown to be glycosylated, each carrying a single N-linked glycan moiety that consists of up to nine hexose residues attached to two core N-acetylhexosamines $(HexNAc)_2$. The masses found in axolotl courtship water point to a similar glycosylation pattern with each glycoprotein set comprising up to four glycoforms (Supplementary Table S1).

In order to compare the measured protein masses to the theoretical relative molecular mass (Mr) of *in silico* translated SPF transcripts, transcriptome data was generated by performing RNA sequencing and RACE-PCR on cloacal tissue of a male that was in breeding condition. Thirteen different SPF cDNA precursor sequences were identified by RACE-PCR sequencing and every translated SPF protein sequence showed at least one potential N-linked glycosylation site (Asn-X-Ser/Thr) (underlined in Fig. 2). A glycosylation pattern similar to the one found in palmate newt SPF proteins was taken into account for the theoretical Mr computation of the mature SPF proteins (Supplementary Table S1)[1]. Since for some SPF precursors multiple N-linked glycosylation sites were predicted, additional calculations were made with two or three N-linked glycan trees.

Three series of glycoforms were identified in courtship water that match the theoretically predicted Mr of three glycosylated SPF isoforms (Supplementary Table S1: SPF3, SPF6 and SPF12/13). All three isoforms bear two HexNAcs and five to

eight hexoses. A fourth set of corresponding glycoform masses could be assigned to one of three above-mentioned proteins as well, but deviates from the theoretically predicted Mr, possibly because of C-terminal cleavage due to carboxy- or endopeptidases in the samples or the existence of similar precursors with an earlier stop codon (Supplementary Table S1: SPF12/13-LLNTLSQ). The order in which the masses and thus their matching SPF cDNA precursors appear in the consecutive RP-HPLC fractions coincides with the order in which the corresponding Edman degradation sequences appear (Table 1 and Supplementary Table S1: Fraction I: SPF12/13, Fraction II: SPF 12/13 and SPF3, Fraction III: SPF12/13, SPF3 and SPF6, Fraction IV: SPF12/13, low mass spectrometry signal for SPF3 and SPF6, but no clear detectable N-terminal sequences anymore). No other masses were deduced from courtship water that could be linked to an SPF precursor for which the N-terminal sequence corresponds to one of the remaining Edman degradation sequences (SPF9, SPF2/5/6/7, SPF4, SPF10/11). Remarkably, all of these precursors show more than one N-glycosylation site, indicating that the isoforms potentially bear more than one N-linked glycan (Fig. 2). An increased variation in glycosylation could hinder mass spectrometry as the existence of many more glycoforms per isoform lowers the individual glycoform concentration and thus ion signal intensity. From fraction V onwards, the mass spectrometry signal became too weak to assign any mass unambiguously, although the silver-stained protein gel still shows a 20-25 kDa band (Fig. 1a, fraction V). This band is significantly less sharp than the bands in fraction I, II and III, which again suggests the existence of additional glycoforms (and thus the presence of more than one N-glycan). No 20-25 kDa masses were measured in female water, while one glycoform set was found in male water (see Supplementary Table S1). The single Edman degradation sequence in male water suggests that these masses

belong to SPF12/13 (Table 1). When taking the possibility of C-terminal degradation into account, all experimentally obtained masses could effectively be linked to a shorter or cleaved form of SPF12/13 (see Supplementary Table S1).

Supplementary Table S1. SPF glycoforms identified by mass spectrometry in courtship and male water. Mass spectrometry data of SPF proteins in courtship water with corresponding cDNA precursors and their theoretically predicted relative molecular mass, taking signal peptide removal, S-S bridge formation and glycosylation into account. TFA adducts (Mr + 114 or 228) were identified for nearly all glycoforms and are not shown. One glycoform series deviates from the theoretically predicted Mr, possibly because of C-terminal degradation due to carboxy- or endopeptidase activity or the existence of similar precursors with an earlier stop codon.

Fraction	Mr measured in courtship water	Mr measured in male water	Protein isoform – Cleaved fragment	Calculated Mr of unglycosylated precursor	HexNAc (Average mass = 203.19)	Hexose (Average mass = 162.14)	Calculated Mr glycosylated precursor
I	21,801.9		SPF12/13	20,584.2	2	5	21,801.3
	21,964.0					6	21,963.5
	22,127.2					7	22,125.6
II	21,801.2		SPF12/13	20,584.2	2	5	21,801.3
	21,963.4					6	21,963.5
	22,128.4					7	22,125.6
		20,708.2	SPF12/13-	19814.3	2	3	20707.1
		20,870.1	LLNTLSQ			4	20869.3
		21,032.3				5	21031.4
		21,194.6				6	21193.6
		21,520.0				8	21517.8
	20,760.6		SPF3	19,544.0	2	5	20,761.1
	20,923.3					6	20,923.2
	21,085.1					7	21,085.4
	21,247.1					8	21,247.5
III	21,801.9		SPF12/13	20,584.2	2	5	21,801.3
	21,964.1					6	21,963.5
	22,125.6					7	22,125.6
		20,707.7	SPF12/13-	19814.3	2	3	20,707.1
		20,870.0	LLNTLSQ			4	20,869.3
	21,032.6	21,032.5				5	21,031.4
	21,192.4	21,194.9				6	21,193.6
	21,354.2					7	21,355.7
	21,516.9	21,520.4				8	21,517.8
	20,761.1		SPF3	19,544.0	2	5	20,761.1
	20,923.6					6	20,923.2
	21,086.2					7	21,085.4
	21,247.3					8	21,247.5
	21,013.6		SPF6	19,796.1	2	5	21,013.2
	21,175.6					6	21,175.3
	21,337.8					7	21,337.4
	21,499.7					8	21,499.6
IV	21,802.0		SPF12/13	20,584.2	2	5	21,801.3
	21,964.2					6	21,963.5
		20,708.9	SPF12/13-	19,814.3	2	3	20,707.1
		20,869.7	LLNTLSQ			4	20,869.3
		21,032.6				5	21,031.4
		21,194.6				6	21,193.6
		21,520.7				8	21,517.8
(only in	20,923.8		SPF3	19,544.0	2	6	20,923.2
fraction	21,247.3					8	21,247.5
54							
(only in	21,175.6		SPF6	19,796.1	2	6	21,175.3
fraction	21,338.7					7	21,337.4
54	21,499.9					8	21,499.6
V	/	20707.9	SPF12/13-	19814.3	2	3	20707.1
		20870.1	LLNTLSQ			4	20869.3
		21032.7				5	21031.4
		21195.0				6	21193.6

Supplementary Table S2. Accession numbers of SPF precursor sequences of species used in the phylogenetic analysis.

Species	Accession number	Tissue	Database
Ambystoma mexicanum 1, 3, 8, 5, 2, 7, 6, 4, 9, 12, 13, 10, 11	KU043451-KU043463		NCBI
Ambystoma mexicanum 14 (*de novo* transcript also in cloaca)	CN035733	larval limb tissue	NCBI
Ambystoma mexicanum 15 (*de novo* transcript also in cloaca)	CN041146	larval limb tissue	NCBI
Ambystoma tigrinum	CN048649	brain tissue	NCBI
Aneides ferreus	AAZ06335	male mental gland	NCBI
Cynops pyrrhogaster 1-2	KU213615-KU213616	male abdominal gland	NCBI
Cynops pyrrhogaster 4-7	KU213618-KU213621	male abdominal gland	NCBI
Cynops pyrrhogaster 15	KU213629	male abdominal gland	NCBI
Cynops pyrrhogaster 25	KU213639	male abdominal gland	NCBI
Desmognathus ocoee	AAZ06329	male mental gland	NCBI
Eurycea guttolineata	AAZ06338	male mental gland	NCBI
Ichthyosaura alpestris 11	KP849572	male abdominal gland	NCBI
Ichthyosaura alpestris 14-15	KP849575-KP849576	male abdominal gland	NCBI
Ichthyosaura alpestris 18	KP849579	male abdominal gland	NCBI
Ichthyosaura alpestris 22	KP849583	male abdominal gland	NCBI
Ichthyosaura alpestris 28	KP849589	male abdominal gland	NCBI
Ichthyosaura alpestris 9	KP849570	male abdominal gland	NCBI
Lissotriton helveticus 1-5	KJ402326-KJ402330	male abdominal gland	NCBI
Lissotriton helveticus 12	KJ402337	male abdominal gland	NCBI
Lissotriton helveticus 8	KJ402333	male abdominal gland	NCBI
Notophthalmus viridescens 54	KP118912	male cloacal tissue	NCBI
Notophthalmus viridescens 57	KP118898	male cloacal tissue	NCBI
Notophthalmus viridescens 64	KP118895	male cloacal tissue	NCBI
Notophthalmus viridescens 81	KP118927	male cloacal tissue	NCBI
Notophthalmus viridescens 96	KM463870	male cloacal tissue	NCBI
Notophthalmus viridescens 31	KP118902	male cloacal tissue	NCBI
Plethodon stormi	DQ097067	male mental gland	NCBI
Pleurodeles waltl 11	KM463932	male cloacal tissue	NCBI
Pleurodeles waltl 16	KM463937	male cloacal tissue	NCBI
Pleurodeles waltl 2	KM463923	male cloacal tissue	NCBI
Pleurodeles waltl 7	KM463928	male cloacal tissue	NCBI
Silurana tropicalis 13	F6PQG9	n.a.	Uniprot
Silurana tropicalis 22	XP_002943341	n.a.	NCBI

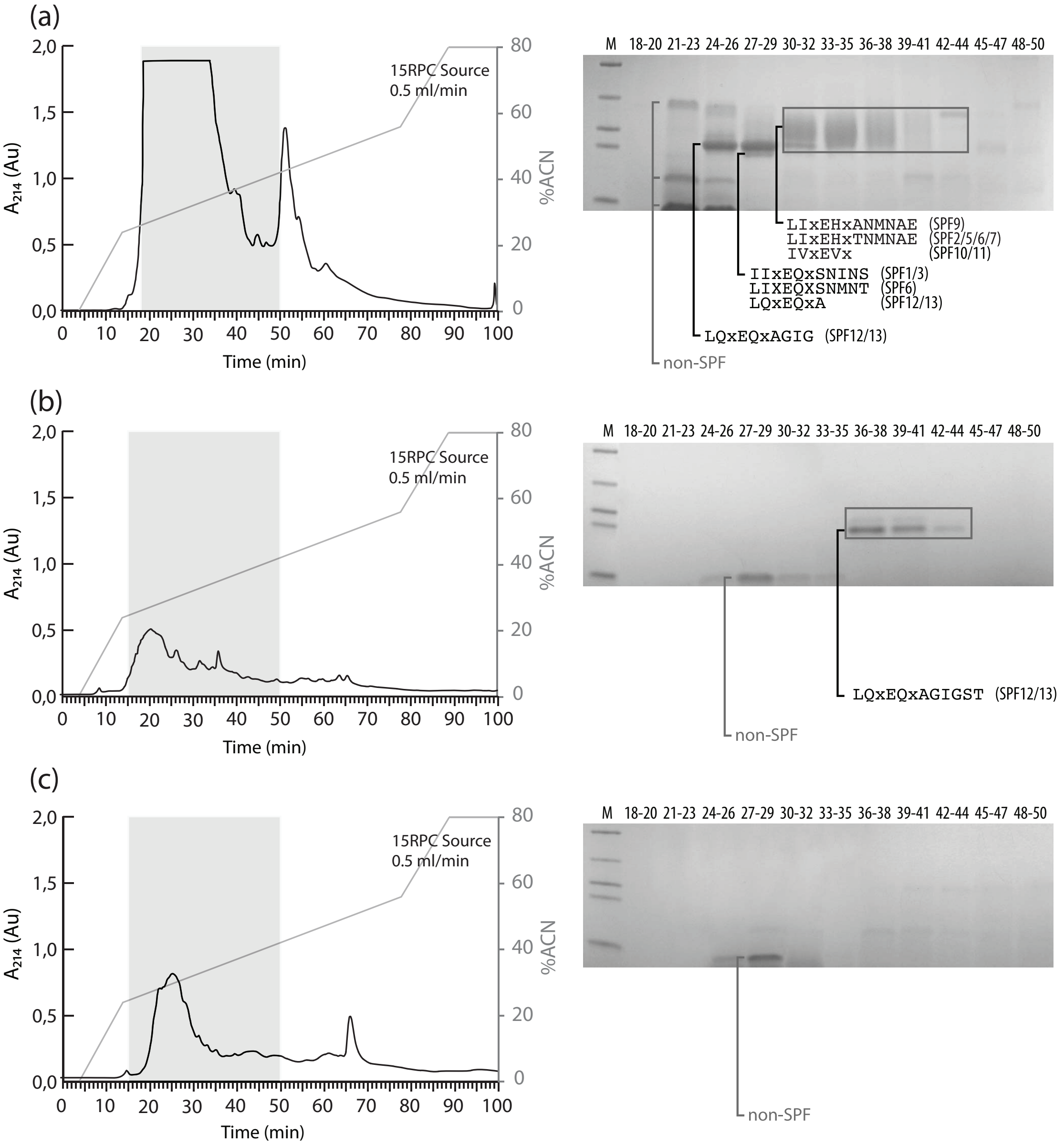

Supplementary Figure S3. Chromatographic separation of proteins present in courtship, male and female water using a 15RPC column. (a) Left: RP-HPLC elution profile of courtship water. Right: courtship water peak fractions ranging from the 18th to 50th minute of the gradient visualized on a silver-stained SDS-PAGE gel (fractions were pooled per three consecutive minutes). Edman degradation sequences of the 20-25 kDa protein bands show the SPF elution range on a 15RPC column and define the fractions to be screened to identify SPF molecules in male and female water. Transcripts obtained by RACE-PCR that correspond to one of

the N-terminal sequences are indicated between brackets. **(b)** Left and right: RP-HPLC elution profile of male water and SDS-PAGE gel of fractions within and surrounding the SPF elution range. Edman degradation sequencing reveals only a single SPF sequence in male water. **(c)** Left and right: RP-HPLC elution profile of female water and SDS-PAGE gel of fractions within and surrounding the SPF elution range. No SPF sequences were identified by Edman degradation sequencing.

References

1. Van Bocxlaer, I. et al. Side-by-side secretion of late Palaeozoic diverged courtship pheromones in an aquatic salamander. *P. Roy. Soc. B-Biol. Sci*. **282**, 20142960 (2015).
